# Supplementary material for: Alcalase-Assisted Mytilus edulis Hydrolysate: A Nutritional Approach for Recovery from Muscle Atrophy
Source: Mar Drugs. 2023 Nov 29;21(12):623. doi: 10.3390/md21120623 (PMC10744518; doi:10.3390/md21120623)
Supplement: Supplementary file 1 [file marinedrugs-21-00623-s001.zip › marinedrugs-2697005-supplementary.pdf]

Supplymentary materials

Materials and methods

1.1 HPLC analysis of AMH

The freeze-dried AMH was reconstituted in distilled water (DW) to achieve a concentration of 100 mg/mL. The solution was then subjected to a 1-hour storage at a temperature of -80 °C, followed by a combination with 0.4% formic acid (FA) in ethyl alcohol at a ratio of 1:9 (v/v). The resulting mixture was subsequently placed in a centrifuge (operating at 4 °C and 13,000 rpm), and the supernatant was carefully collected. This collected supernatant underwent an evaporation process using a stream of nitrogen gas until complete dryness was achieved. After achieving complete dryness, the sample was introduced into a solution comprising 1 mL of 4% H_3PO_4 in DW (v/v). This solution was then applied to an OASIS MAX® Solid Phase Extraction (SPE) column from Waters (Ireland), following the recommended 'OASIS PST Protocol' provided by the manufacturer. In a concise overview of the process, the SPE column was sequentially subjected to two equilibration solutions: firstly, 100% methanol, and secondly, DW. The initial equilibration was followed by loading the sample onto the SPE column. Subsequently, the sample loaded SPE column underwent two distinct washing steps: the first wash involved 5% NH_4OH, while the second wash employed a solution containing 20% acetonitrile (ACN). Upon completing these washing steps, the purified sample was eluted using a solution containing 75% ACN in DW, augmented with 1% FA. The chromatographic separation was carried out using a poroshell 120 EC-C18 column (4.6 mm × 100 mm, 4.0 µm, Agilent, USA). The injection volume was 10 µL and the detection was performed at 280 nm wavelength using UV-Vis detector. UPLC elution step was performed under these conditions: 100% B for 5 min, 50% B up to 60 min, 10% B up to 80 min, 100% B up to 95 min. followed by 10 minutes of column re-equilibration.

Supplementary Figure Legends

Figure S1. HPLC analysis of the AMH (100 mg/mL). (a) Blank (b) Fraction-1: AMH in 5% Ammonium 241 water (NH4OH) (c) Fraction-2: AMH in 20 % Acetonitrile and (d) Fraction-3: AMH in 1% Formic 242 acid. A UV detector was used for the analysis and the detection wavelength was 280 nm

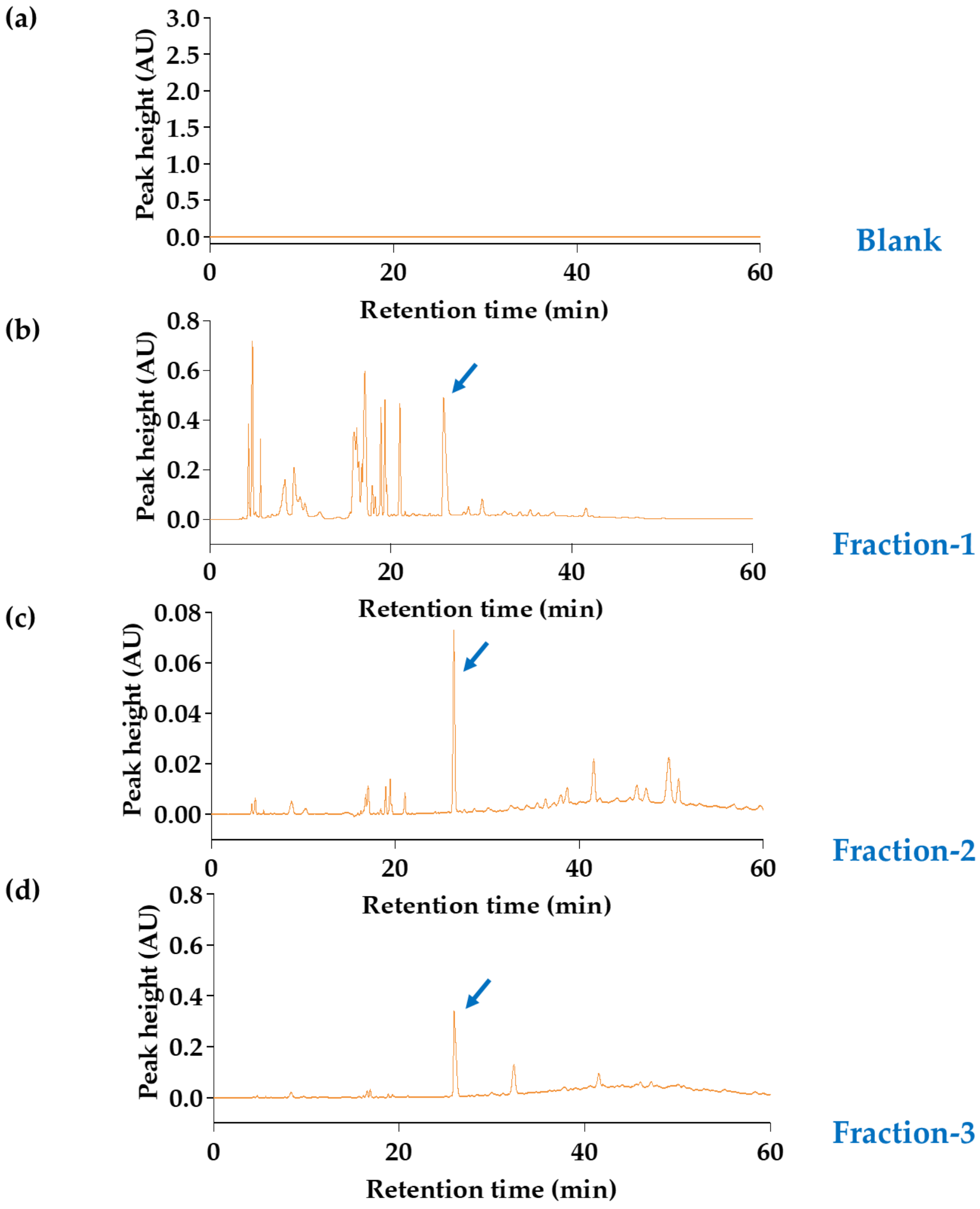

(a)
Peak height (AU)
3.0
2.5
2.0
1.5
1.0
0.5
0.0
0
20
40
60
Retention time (min)
Blank
(b)
Peak height (AU)
0.8
0.6
0.4
0.2
0.0
0
20
40
60
Retention time (min)
Fraction-1
(c)
Peak height (AU)
0.08
0.06
0.04
0.02
0.00
0
20
40
60
Retention time (min)
Fraction-2
(d)
Peak height (AU)
0.8
0.6
0.4
0.2
0.0
0
20
40
60
Retention time (min)
Fraction-3

36
